# Microarray-Based Detection and Clinical Evaluation for* Helicobacter pylori* Resistance to Clarithromycin or Levofloxacin and the Genotype of CYP2C19 in 1083 Patients

**DOI:** 10.1155/2018/2684836

**Published:** 2018-09-10

**Authors:** Yi Song, Fengna Dou, Zhe Zhou, Ningmin Yang, Jing Zhong, Jie Pan, Qiqi Liu, Jianzhong Zhang, Shengqi Wang

**Affiliations:** ^1^Beijing Institute of Radiation Medicine, Beijing, China; ^2^Beijing Key Laboratory of New Molecular Diagnosis Technologies for Infectious Diseases, Beijing, China; ^3^Hangzhou Zhiyuan Medical Laboratory Co., Ltd., Hangzhou, China; ^4^Huzhou Central Hospital, Huzhou, China; ^5^Wenzhou Central Hospital, Wenzhou, China; ^6^State Key Laboratory of Infectious Disease Prevention and Control, Collaborative Innovation Center for Diagnosis and Treatment of Infectious Diseases, Chinese Center for Disease Control and Prevention, Beijing, China

## Abstract

*Background. Helicobacter pylori* (*H. pylori*) is one of the most frequent and persistent bacterial infections that affect nearly half of the world's population. Antibiotic resistance is a constantly evolving process and local surveillance of antibiotic resistance is warranted to guide clinicians in their choice of therapy. The aim of this study was to establish a microarray-based detection to identify* H. pylori* infection, clarithromycin and levofloxacin susceptibility, and CYP2C19 genetic polymorphism and guide to potential choice of proton pump inhibitor (PPI), antibiotic administration for tailored* H. pylori* eradication therapy.* Methods.* By analyzing the sequence of human genomic* CYP2C19*⁎*2* and* CYP2C19*⁎*3* and mutations within the* 23S rRNA *and* gyrA* gene regions conferring clarithromycin and levofloxacin resistance, respectively, we developed a microarray for individual therapy detection of* H. pylori* infection. Plasmids were established as positive or limit of detection (LOD) reference materials. The specificity and sensitivity of the microarray had been performed. And a total of 1083 gastric biopsy samples were tested and the Kappa value had been calculated between the array and Sanger sequencing. We also analyzed the resistance to clarithromycin and levofloxacin in China, as well as the CYP2C19 polymorphisms.* Results.* The LOD of detecting* H. pylori* was 10^3^ CFU/mL and human genome DNA was 2 ng/*μ*L. The detection results of 1083 gastric biopsy samples showed that 691 (63.80%) were* H. pylori *positive, of which 266 (38.49%) were resistant to clarithromycin, 192 (27.79%) were resistant to levofloxacin, and 61 (8.83%) were resistant to both of them. For the type of CYP2C19 polymorphism, 412 (38.04%) were homozygous fast type (HomEM), 574 (53%) were heterozygous EM (HetEM), and 97 (8.96%) were poor metabolizer (PM).* Conclusions.* The proposed microarray-based detection has high specificity, sensitivity, and reproducibility for detecting the resistance of clarithromycin or levofloxacin as well as CYP2C19 polymorphism, which may help to improve the clinical eradication rate of* H. pylori*.

## 1. Introduction

More than 50% of the world's population is infected with* Helicobacter pylori (H. pylori)*, and the rate of infection among Chinese adults is 40%-60% [1].* H. pylori *infection is associated with a variety of gastrointestinal diseases such as gastritis, peptic ulcer and promotes the accumulation of various mutations and might promote gastric carcinogenesis [2].

At present, triple or quadruple therapy based on proton pump inhibitors (PPIs) combined with amoxicillin and clarithromycin (or metronidazole) is recognized as a first-line treatment of* H. pylori*. PPI-clarithromycin-amoxicillin with a bismuth quadruple regimen was recommended as the second-line after prior treatment failure in the Maastricht V/Florence Consensus Report [3] and the Fifth Chinese Consensus Report [1]. The efficacy of therapy is mainly affected by the following two factors: one hand is antibiotic resistance and primary drug-resistance rate of three out of six kinds of antibacterial drugs recommended by consensus report has raised, metronidazole (40%-70%), clarithromycin (20%-50%), and levofloxacin (20%-50%), and even multidrug resistance also occurred. The antibiotic resistance has significantly reduced the eradication rate [1, 4, 5]. Due to resistance, eradication rate of standard triple therapy has less than the 80%, and even the treatment time of standard triple therapy increased from 7d to 10d or 14d, and eradication rate can only improve about 5% [6]. Another hand is that PPI inhibitors pay an important role for the success of triple therapy. PPIs are serials of pharmaceutical agents that target H^+^/K^+^-ATPase and have high potency to increase gastric pH value coupled with minor side effects, which made them very popular in gastrointestinal disorders, particularly in* H. pylori *infection eradication regimen [7]. PPIs' high acid suppression make acid-labile antibiotics such as clarithromycin more stable by increasing gastric pH value, thereby increasing concentration and* H. pylori *sensitivity to antibiotics [8]. PPIs (except rabeprazole) are mainly metabolized by cytochrome P450 2C19 (CYP2C19), whose highly polymorphic genotype can cause variability response in omeprazole- based or lansoprazole-based triple therapies [9].* CYP2C19∗2 *(681 G > A, rs4244285) and* CYP2C19∗3* (636 G > A, rs4986893) are most frequent variant and the two types alleles associated with loss of function [10]. Other known factors that contributed to the increased failure rate of* H. pylori* eradication include poor compliance, high gastric acidity, and high bacterial load [11].

According to* CYP2C19∗2 *and* CYP2C19∗3,* the CYP2C19 phenotype has been classified into three groups: homozygous extensive metabolizers (Hom-EMs), heterozygous extensive metabolizers (Het-EMs), and poor metabolizers (PMs) [12]. Research shows that, in the first-line treatment based on PPI, PM patients can get the highest* H. pylori* eradication rate. In remedial therapy based on PPIs, the* H. pylori *eradication rate between HomEM and HetEM is significantly different [7].

There are four most often observed point mutations associated with clarithromycin: A2144G, A2143G, A2142G, and A2143C in* 23S rRNA* [12]. “Hot-spots” (A2142G and A2143G, approximately 80% of all mutations) domain V has shown the major mechanism of clarithromycin resistance [11, 13]. Mutations at amino acid 87 (Asn to Lys, Tyr, or Ile) and/or 91 (Asp to Asn, Gly, or Tyr) in the quinolones resistance-determining region (QRDR) of* gyrA *weakened binding between the quinolone antibiotics and the gyrase, causing antibiotic resistance [14–16]. Mainly Asn-87-Lys and Asp-91-Gly were found in 92.8% (52/56) of the levofloxacin-resistant isolates [17].

In this study, we established a visual DNA microarray method to detect susceptibility of clarithromycin and levofloxacin, polymorphisms of CYP2C19 in patients simultaneously. After validating accuracy, specificity, and sensitivity of the microarray, we applied it in clinical research by detecting gastric mucosa samples of 1083 cases and assessed the antibiotic resistance of* H. pylori* and genetic polymorphism of CYP2C19 in China.

## 2. Materials and Methods

### 2.1. Specimen Collection and Processing

The gastric biopsy samples were collected from Chinese Center for Disease Control and Prevention (342), Chinese Huzhou Central Hospital (394), and Chinese Wenzhou Central Hospital (347), respectively. Of the subjects, 541 were male patients (49.95%) and 542 were females (50.05%), with ages ranging from 14 to 85 years. Subjects undergoing gastroscopy and sampling for diagnosis of upper gastrointestinal diseases were recruited. A total of 660 patients were positive for 13C/14C-urea breath test. Of these patients, 403 without a history of treatment for* H. pylori* infection and 257 had previously received* H. pylori* eradication therapy.

Gastric biopsy specimens were crushed or cut into pieces using a sterile glass homogenizer by the Full Automatic Tissue Grinding Instrument (Tuhe Mechatronics Co., Shanghai, China) and mixed with 200 *μ*L normal saline. Total DNA mixtures from biopsy specimens were extracted using the QIAmp DNA Mini Kit (Qiagen, Hilden, Germany) following the protocol.

### 2.2. Multiplex PCR

DNA regions involved in clarithromycin (*23S rRNA*), quinolone (*gyrA*) resistance,* CYP2C19∗2* (681 G > A, rs4244285), and* CYP2C19∗3 *(636 G > A, rs4986893) were amplified by PCR as previously reported [18–20]. Primers were picked in the conserved upstream or downstream regions and probes were designed at positions of mutations (Table 1). All the primers and probes were verified by BLAST (http://blast.ncbi.nlm.nih.gov/).

All reverse primers for target genes were labeled by biotin at the 5′-ends and multiplex PCR was performed in one tube. Reaction mixtures (25 *μ*L) contained 12.5 *μ*L of 2× Multiplex PCR Mix (cwBiotech, Beijing, China) and 3 *μ*L of DNA template. The concentrations of forward and reverse primers all were 0.12 *μ*M and 1.2 *μ*M, respectively. PCR was performed on a Thermal Cycler PCR system (Applied Biosystems, Foster City, US) using the following conditions: 15 min at 95°C; 45 cycles of 30 s at 94°C, 60 s at 65°C, and 30 s at 72°C; and a final extension of 5 min at 72°C.

### 2.3. Construction of Reference Plasmids

The international standard strain* H. pylori* 26695 was used to construct the wild-type control plasmid and normal human genomic DNA was used as template for PCR amplification. Mutant control plasmids were constructed using PCR site-directed mutagenesis to amply fragments of mutations in specific sites. The purified amplification products were ligated with PGM-T vector and cloned into DH5*α*. The target fragments were identified by sequencing the entire regions. Then the positive clones were picked to culture. After the determination of the initial concentration, the bacteria liquid was diluted to 1 × 10^5^, 1 × 10^4^, 1 × 10^3^, and 1 × 10^2^ CFU /ml by using phosphate buffer saline. Plasmids of human genomic DNA were extracted and diluted to 10 ng/*μ*L, 5 ng/*μ*L, 2 ng/*μ*L, and 1 ng/*μ*L in order to determine the LOD of the microarray.

### 2.4. Microarray Fabrication

**Figure 1:**
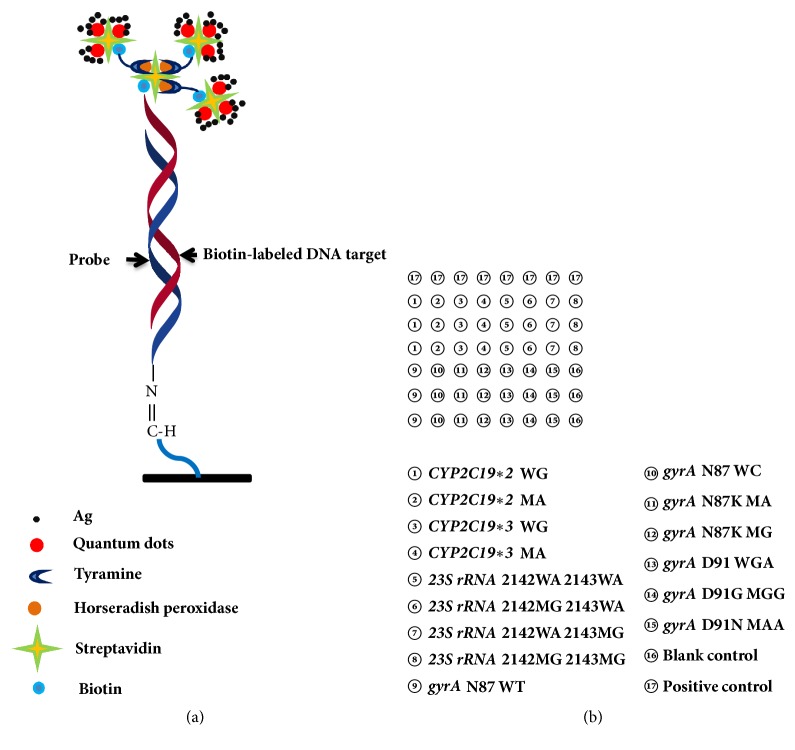
(a) Schematic diagram of the principle of microarray visualization technology based on tyramine signal amplification coupled with quantum dot-catalyzed silver deposition. (b) The microarray layout of* CYP2C19∗2*,* CYP2C19∗3*,* 23S rRNA*, and* gyrA *genes that are detected in this study; W: wild genotype; M: mutation genotype.

A repeat sequence of 12T- with an amino labeled 3′-end was connected to the 3′-end of all the probes and fixed on the aldehyde-chip surface. All modified microarray probes were synthesized by Sangon Biotech Co., Ltd. (Shanghai). Probes, at 50 *μ*M final concentration, were spotted thrice repeatedly by using a noncontact inkjet Nano-plotter 2.1 (GeSim, Dresden, Germany) on the aldehyde-chip after mixing with uniform proportional printing buffer (5% glycerol, 0.1% sodium dodecyl sulfate (SDS), 6×saline-sodium citrate buffer (SSC), and 2% (wt/vol) Ficoll 400). Quality control (QC) probe was spotted eight times repeatedly in the horizontal direction at a final concentration of 12.5*μ*M in order to manage the standard operation and calibrate the signal values. Each aldehyde slide was divided into 10 blocks (11 × 11 mm) by a waterproof film that can detect 10 different samples. The layout was shown in Figure 1(a) and the principle of hybridization was shown in Figure 1(b). The fabricated microarray was placed in a dryer for 24 h at room temperature. By washing once with 0.2% SDS and once with distilled water for 30 s at room temperature, the unbound probes were removed before use.

### 2.5. Hybridization and Signal Detection

After amplification, the products were denatured at 95°C for 5 min and immediately placed on ice for 5 min and then mixed with 5 *μ*L of hybridization buffer (8× SSC, 0.6% SDS, 10% formylamine, and 10× Denhardt). A total of 10 *μ*L hybridization mixture were hybridized on the microarray in a hybrid-box for 45 min at 45°C. The slide was washed for 30s successively in 1× SSC and 0.2% SDS, 0.2× SSC, and 0.1× SSC at room temperature. Then the chip was incubated with 15 *μ*L of 25 nM streptavidin-quantum dots (StrQDs, Wuhan Jiayuan Quantum Dots Co., Ltd.) for 30 min at 37°C and washed with PBS-T (phosphate buffer, 0.05% Tween 20) five times for 20 s and distilled water once for 10 s at room temperature. Subsequently, 30 *μ*L of aqueous silver acetate solution and hydroquinone citric acid solution (each 15 *μ*L) mixture were added. When the black signal point appeared, the chip was washed with distilled water and dried to scan by Image Scanner (UMAX, Amersham Biosciences). The probe signal densities were calculated by Gel-Pro Analyzer Vision 4.0 (Media Cybernetics, Silver Spring, USA).

### 2.6. Confirmation by Sequencing and Statistical Analysis

All these 1083 gastric biopsy samples were characterized by PCR amplification and gel electrophoresis. PCR-amplified fragments were purified by using DNA purification Kit DP241 (Tiangen Biotech Beijing Co., Ltd.) and sequenced by the dideoxy chain termination method.

According to the sequencing results, the coincidence rate of the microarray results had been calculated. McNemar Chi-square test and Kappa consistency check were performed to identify the difference and consistency between the microarray and the sequence. The SPSS 17.0 software package (SPSS Inc. Chicago, USA) was used for all analyses.

## 3. Results

### 3.1. Determination of Threshold Signal Intensity

The signal intensities values were quantified by Gel-Pro analyzer and the signal intensities were calibrated as follows: the calibrated value of a probe = mean signal intensities value of the probe / mean signal intensities value of the QC probe (the same detection block) ×100.

For the two probes of* CYP2C19 ∗2*, the ratio of the calibrated value of 2C19 *∗* 2W to that of 2C19 *∗* 2 M was determined. If the ratio was greater than 2.0, the locus was considered to be wild type and homozygote, while if the ratio was less than 0.5, the loci were considered to be homozygous mutation. If the ratio was between 0.5 and 2.0, we determined this locus was heterozygous. Two probes of* CYP2C19 ∗ 3* (2C19 *∗*3W and 2C19 *∗* 3M) criteria were the same as* CYP2C19 ∗ 2*. For the four probes of 23S rRNA (42W43W, 42M43W, 42W43M, and 42M43M), the one of calibrated value two times greater than the other three ones was considered to be positive. Criteria of the four 87-locus probes (87WT, 87WC, 87MA, and 87MG) and three 91-locus probes (91W, 91MG, and 91MA) of* gyrA *were the same as* 23S rRNA*.

### 3.2. Specificity and Sensitivity of Microarray Test

**Figure 2:**
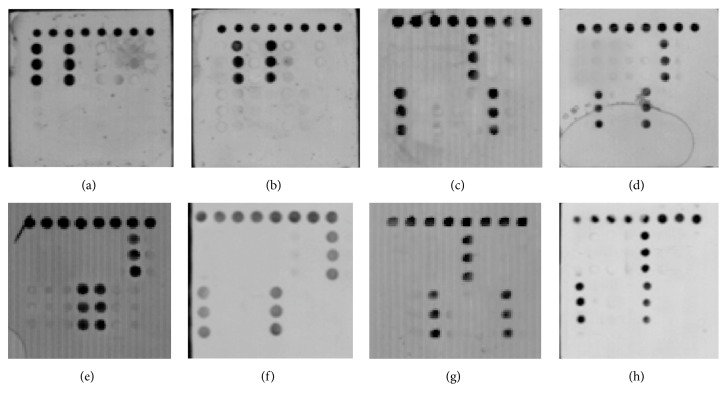
Visible detection of* CYP2C19∗2*,* CYP2C19∗3*,* 23S rRNA*, and* gyrA* (positive and negative controls). (a) Wild genotype of* CY2C19∗2* and* CY2C19∗3*. (b) Mutation genotype of* CY2C19∗2* and* CY2C19∗3*. (c) Wild at position 2142 and 2143 of* 23S rRNA*, wild genotype T at Asn-87, and mutant genotype GG at Asp-91 of* gyrA.* (d) Mutant at position 2142 and wild at position 2143 of* 23S rRNA*, wild genotype C at Asn-87 and wild genotype GA at Asp-91 of* gyrA*. (e) Wild at position 2142 and mutant at position 2143 of* 23S rRNA, *mutant genotype G at Asn-87, and wild genotype GA at Asp-91 of* gyrA*. (f) Mutant at position 2142 and 2143 of* 23S rRNA*, wild genotype T at Asn-87, and wild genotype GA at Asp-91 of* gyrA*. (g) Wild at position 2142 and 2143 of* 23S rRNA, *mutant genotype A at Asn-87, and mutant genotype AA at Asp-91 of* gyrA*. (h) Standard strain 26695 of* H. pylori.*

To evaluate the specificity of the microarray method, we detected the reference plasmids. The result images covering 15 probes were shown in Figure 2 and showed that the microarray was able to exactly distinguish the mutation sites of these nucleotides. The negative control* H. pylori *26695 showed the negative microarray results which also demonstrated the specificity of this assay (Figure 2).

**Figure 3:**
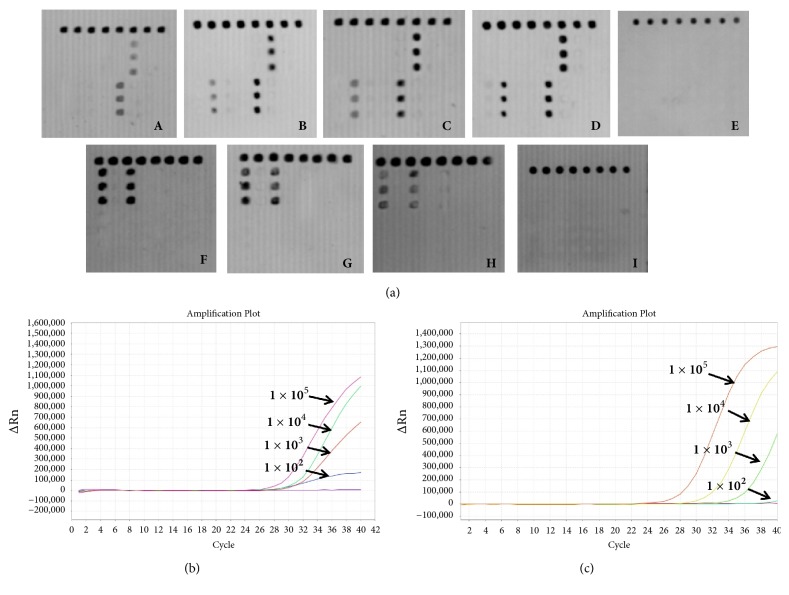
(a) Sensitivity of the microarray assays. A ~ D were four dilutions from 1 × 10^2^ to 1 × 10^5^ CFU/mL for plasmid bacteria and E was blank control, F ~ I were 10 ng /*μ*L, 5 ng /*μ*L, 2 ng /*μ*L, and 1 ng /*μ*L for human genomic DNA. (b)* 23S rRNA *reference plasmids (1 × 10^2^ to 1 × 10^5^ CFU/mL) were detected by the real-time PCR and (c)* gyrA* reference plasmids (1 × 10^2^ to 1 × 10^5^ CFU/mL) were detected by the real-time PCR.

To determine the absolute LOD of this strategy, serially diluted recombinant plasmids had been quantified by the copy numbers served as the reference materials (i.e., from 1 ×10^5^, 1 × 10^4^, 1 × 10^3^, and 1 × 10^2^ CFU/mL for plasmid bacteria and 10, 5, 2, and 1 ng /*μ*L for human genomic DNA). The absolute LODs for the plasmids were 10^2^ CFU/mL and 2 ng /*μ*L for the human genomic DNA (Figure 3(a)).* 23S rRNA* and* gyrA* of* H. pylori* detection kit (PCR-Fluorescence Probing, provided by our laboratory) were also used to detect* 23S rRNA* and* gyrA* reference plasmids (1 × 10^1^ to 1 × 10^5^ copies/*μ*L), respectively. The results of sensitivity comparison between microarray assay and real-time PCR showed that they had similar sensitivities (Figures 3(b) and 3(c)).

### 3.3. Detection of Human* CYP2C19 *Gene Polymorphism

**Figure 4:**
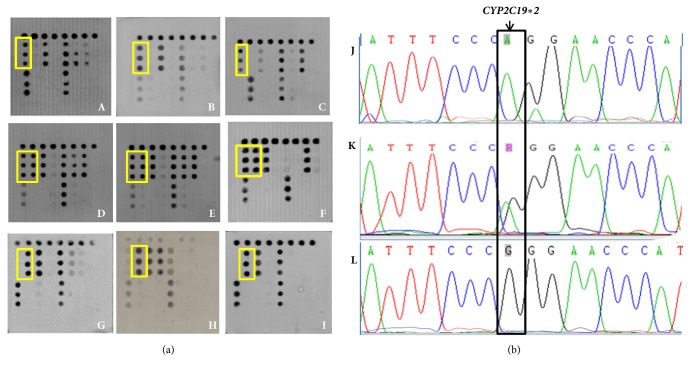
(a)* CYP2C19∗2* detection results of the microarray (indicating by yellow frame): A, B, and C were wild genotype GG; D, E, and F was heterozygous genotype AG/GA; G, H, and I were mutation genotype AA. (b)* CYP2C19∗2* detection results of sequencing (indicating by black frame): J was homozygous AA; K was heterozygous AG/GA; L was homozygous GG.

**Figure 5:**
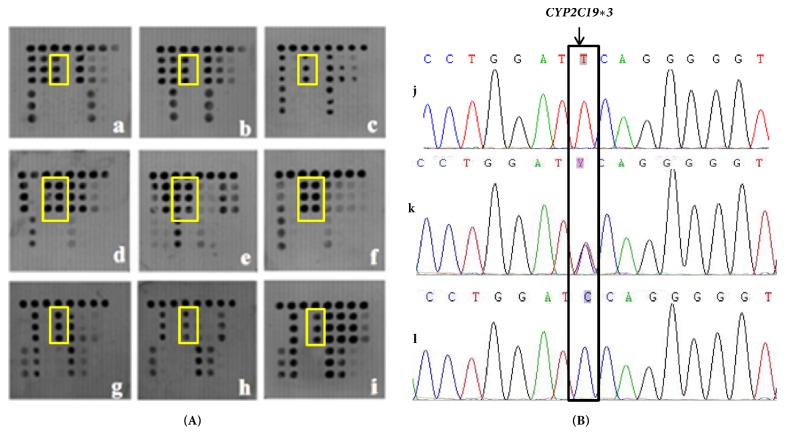
(A)* CYP2C19∗3* detection results of the microarray (indicating by yellow frame): a, b, and c were wild genotype GG; d, e, and f were heterozygous genotype AG/GA; g, h, and i were mutation genotype AA (B)* CYP2C19∗3* detection results of sequencing (indicating by black frame): j was homozygous AA(RC); k was heterozygous AG/GA(RC); l was homozygous GG (RC).

The CYP2C19 polymorphic genotype in all 1083 patients was determined by microarray-based assay coupled with nucleotide sequencing. The results of the microarray showed that 412 cases (38%) were HomEM genotype for harboring two wild-type alleles on* CYP2C19∗2* and* CYP2C19∗3* loci. There were 574 cases (53%) that had one loss-of-function (LOF) variant allele in* CYP2C19∗2* or* CYP2C19∗3* that compromised the rates of PPI metabolism which were HetEM. PM carries two LOF variant alleles (*CYP2C19∗2* and* CYP2C19∗3*) and 97 (9.0%) were PM in our study. Part of the microarray and sequencing results are shown in Figures 4 and 5. Compared with Sanger sequencing, the sensitivity, specificity, and concordance was 97.68%, 93.75%, and 95.94%, respectively for* CYP2C19∗2 *(Table 2) and 81.36%, 99.90%, and 98.89%, respectively, for* CYP2C19∗3* (Table 3). We performed a McNemar test, which indicated that both methods provide equivalent results (McNemar chi-square* *=* *1.885,* P *=* *1.000 > 0.05 for* CYP2C19∗2* and chi-square* *=* *1.906,* P *=* *1.000 > 0.05 for* CYP2C19∗3*). Kappa consistency test showed that Kappa=0.929(*P* < 0.0001) for* CYP2C19∗2* and Kappa=0.884 (*P *< 0.0001) for* CYP2C19∗3*, which revealed that the consistency between microarray and sequencing was statistically significant.

### 3.4. Detection of* 23S rRNA* and* gyrA* Mutations

**Figure 6:**
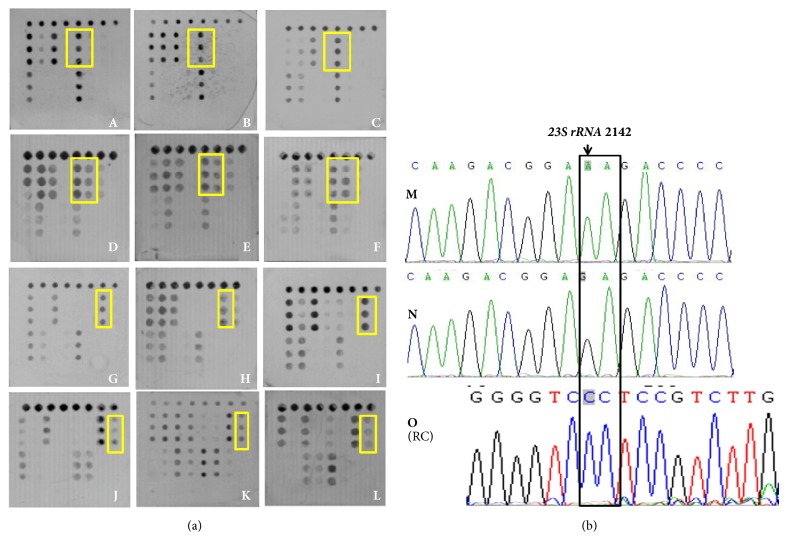
(a) Microarray detection results of* 23S rRNA *(indicating by yellow frame): A, B, and C were wild genotype AA; D, E, and F were heterozygous at 2142 and wild at 2143, the genotype was GA; G, H, and I were wild at 2142 and mutant at 2143, the genotype was AG; J, K, and L were mutation genotype GG. (b) Sequencing results of* 23S rRNA *(indicating by black frame): M was wild genotype AA; N was mutation at 2142 and wild at 2143, the genotype was GA; O was mutation genotype GG (RC: reverse complementary sequencing).

Determined by sequencing, there were 691 cases as* H. pylori* positive and 266 cases that showed mutations of* 23S rRNA*. Part of the microarray and sequencing results are shown in Figure 6. The sensitivity, specificity, and concordance were 92.13%, 93.75%, and 95.94%, respectively, between the microarray and sequencing (Table 4). The performance of the two methods is not significantly different (chi-square = 5.897,* P*=1.000 > 0.05; Kappa value was 1.000,* P *< 0.0001).

**Figure 7:**
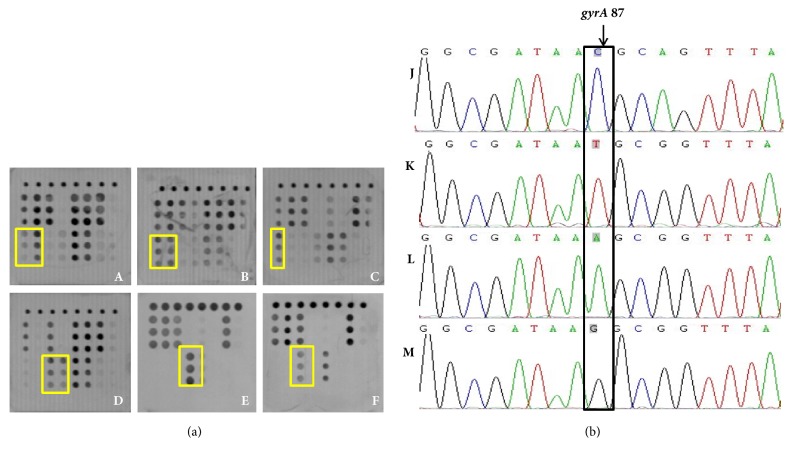
(a) Microarray detection results of* gyrA *at Asn-87 (indicating by yellow frame): A, B, and C were wild genotype T/C; D, E, and F were mutation genotype A/G. (b) Sequencing results of* gyrA *at Asn-87 (indicating by black frame): J was wild genotype C, K was wild genotype T, L was mutation genotype A, and M was mutation genotype G.

**Figure 8:**
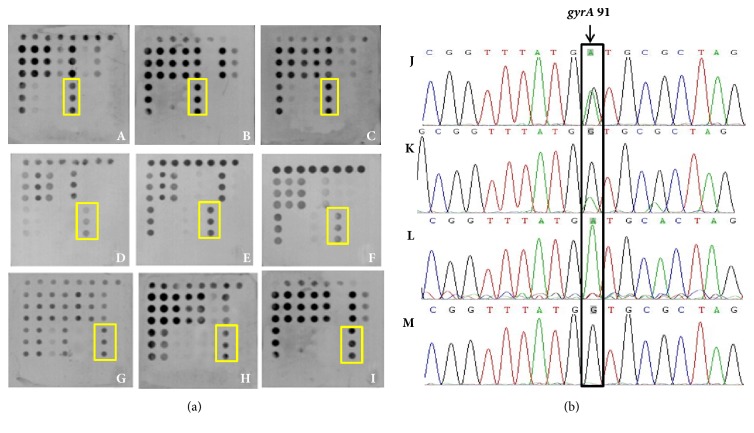
(a) Microarray detection results of* gyrA *at Asp-91 (indicating by yellow frame): A, B, and C were wild genotype GA; D, E, and F were mutation genotype GG; G, H, and I were mutation genotype AA. (b) Sequencing results of* gyrA *at Asp-91 (indicating by black frame): J and K were wild genotype GA, L was mutation genotype AA, and M was mutation genotype GG.

For QRDR of the* gyrA* gene, 192 samples had point mutations in which 135 were at amino acid Asn-87 and amino acid Asp-91 were at codon 91. The sensitivity and specificity of microarray-based detection at Asn-87 were 91.11% and 98.20% and at Asp-91 were 91.23% and 98.90%. The consistency rate for codon 87 was 96.82% and for codon 91 was 98.26% between the microarray and sequencing (Tables 5 and 6). Part of the microarray and sequencing results are shown in Figures 7 and 8. For statistical analysis, the chi-square was 5.575 (*P*=1.000 > 0.05) and Kappa value was 0.898 (Z=23.61,* P* < 0.0001) at codon 87 of* gyrA* and chi-square was 5.439 (*P*=1.000 > 0.05) and Kappa value was 0.887 (Z=23.323,* P *< 0.0001) at codon 91, indicating there is no statistical difference between the results of microarray and sequencing.

### 3.5. Correlation between* H. pylori* Mutations and CYP2C19 Genetic Polymorphism

Table 7 showed a correlation between the mutations of* H. pylori *and CYP2C19 genetic polymorphism. Chi-square test was used to assess differences and there was no significant correlation that was observed between CYP2C19 genetic polymorphism and mutations of* H. pylori* to clarithromycin (*P* = 0.079) and levofloxacin (*P*=0.17).

## 4. Discussion

In China, prevalence of* H. pylori* strains resistance to clarithromycin, metronidazole, and levofloxacin (fluoroquinolone) has been increasing over the last decades. Studies show that clarithromycin resistance was higher than 10% and the success of standard eradication (first-line therapy) was less than 85% [18]. Many studies had confirmed that the eradication rate of clarithromycin sensitive strains was 87%-92%, while the eradication rate decreased to 18%-21% in clarithromycin resistant strains [19]. Levofloxacin was proposed as a salvage treatment regimen after the failure of clarithromycin-based treatments [20]. However, the frequency of levofloxacin resistance was widely recognized throughout the world, which may reduce the efficacy of levofloxacin-based therapeutic regimen [21, 22]. On the whole, the resistance rate of these antibiotics was every high in China; however, regional differences were exit. On the contrary, the resistance rates of* H. pylori* to amoxicillin (0%~5%), tetracycline (0%~5%), and furazolidone (0%~1%) were still very low [23–25].

By assessing sensitivity of clarithromycin, levofloxacin, and genotypes of CYP2C19 before using the triple therapy, the choice of drugs for individual medication can ensure the success rate of treatment and reduce the overuse of antibiotics adverse reaction and waste of resources.

In this study, we designed specific probes to DNA regions involved in clarithromycin (*23S rRNA*), levofloxacin (*gyrA*) resistance,* CYP2C19∗2* (681G>A, rs4244285), and* CYP2C19∗3* (636G>A, rs4986893). The DNA microarray could be used to provide a type of antibiotics sensitivity of clarithromycin, levofloxacin, and polymorphisms of CYP2C19 before the patients' treatment with triple or quadruple therapy. The microarray was fast so the entire experiment could be finished within 6 h from DNA extraction of samples to obtain visual results by naked eyes. The specificity and sensitivity of the microarray had been validated: the specificity concordance was 97.49% compared with the sequencing, and the sensitivity was 10^3^CFU/ml equivalent to fluorescence quantitative PCR (Figure 3).

DNA microarray technology was high throughput, efficiently, and low cost. Quantum dots served as a semiconductor nanocrystalline material due to their good fluorescence characteristics [23], which was widely used in bioimaging. Quantum dots acted as similar electron transfer and could convert a silver ion to a metallic silver atom in the presence of reductive reagents (such as hydroquinone). Silver atom deposited on the surface of quantum dots and could further catalyze the conversion of silver around. The cascade catalysis made silver particles accumulate together and form into silver shell, and then formation of black particles was visible by naked eyes. In this study we developed a new microarray visualization technology based on tyramine signal amplification coupled with quantum dot-catalyzed silver deposition (TSA-QDS) to detect and evaluate* H. pylori* resistance to clarithromycin or levofloxacin and the genotype of CYP2C19. The TSA-QDs possessed similar detection sensitivity as real-time PCR of 10^3^ CFU/mL [26, 27] and the signal point of the method can be scanned by general scanners which are less expensive than fluorescence scanner. Without equipping sophisticated and expensive scanner, the microarray visualization technology greatly reduces the cost (less than six dollars per sample) of chip technology platform to popularize. Compared with traditional methods to detect gastric biopsy specimens such as phenotypic culture [28] and real-time PCR, the method was high throughput and accessible to detect coinfections with clarithromycin and/or levofloxacin-resistant and susceptible strains.

This study showed that distribution of CYP2C19 gene polymorphism in population from both Beijing in northern China and Zhejiang province in south eastern China was consistent with that of Chongqing in southwestern China [24] and Asia [7]. PMs and EMs accounted for a significant proportion in Asian population, while EMs accounted for one in Europeans [25]. CYP2C19 polymorphism could influence the PPIs metabolization and H^+^ concentration in stomach and thus affect activity and stability of antibiotics.* H. pylori* was not completely destroyed or inhibited to grow by these antibiotics in the microenvironment, which might lead to microevolution of* H. pylori* even development of drug resistant strains [24]. Our study indicated that CYP2C19 genetic polymorphism was not significant associated with susceptibility of clarithromycin (*P* = 0.079) or levofloxacin (*P*=0.17); however there was a trend of correlation between CYP2C19 genotypes and clarithromycin resistance. When comparing the* 23S rRNA* mutations in CYP2C19 EMs and CYP2C19 PMs, the number of mutations was significantly higher in the CYP2C19 EM than in the CYP2C19 PM (*P*=0.037 < 0.05).

This study has some limitations. Firstly, the sequencing results of some samples were not consistent with the results of microarray detection. The false negative results of microarray may be due to the difference of the PCR amplification system. For microarray detection, all target genes were amplified in one tube while single PCR amplification was performed for sequencing, which may reduce the sensitivity of microarray detection. Secondly, the DNA microarray could not cover all genes of antibiotics for the eradication of* H. pylori*. Therefore, future studies could address the genes that account for antibiotic resistance of* H. pylori*.

## 5. Conclusions

In summary, we established a visible microarray-based detection that could simultaneously and specially determine the A2142G and A2143G mutation associated with clarithromycin, mutations at amino acids 87 and 91 in the levofloxacin, and human CYP2C19 genotype. The microarray-based detection was rapid, reliable, and high throughput and could be used easily for clinical applications in guidance of individual* H. pylori* eradication therapy.

## Figures and Tables

**Table 1:** The primer and probe sequences for microarray.

Targeted gene	Primer/Probe	Sequencing (5'-3')
CYP450	2C192-F^a^	CAGAGCTTGGCATATTGTATCT
	2C192-R ^b^	GATCAGGAAGCAATCAATAAA
	2C193-F	GATCAGCAATTTCTTAACTTGATGGA
	2C193-R	TTCAAAAATGTACTTCAGGGCTTGGT
	2C192W ^c^	ATTATTTCCCGGGAACCCAT
	2C192M ^d^	TTATTTCCCAGGAACCCAT
	2C193W	TAAGCACCCCCTGGATCCAGGT
	2C193M	GTAAGCACCCCCTGAATCCAGG

*23S rRNA*	H. PYLORI23S-F	ATTCAGTGAAATTGTAGTGGAGGTGA
	H. PYLORI23S-R	CCATTAGCAGTGCTAAGTTGTAGTA
	42W43W	CGGCAAGACGGAAAGACCCC
	42M43W	CGGCAAGACGGGAAGACCCC
	42W43M	GGCAAGACGGAGAGACCCCG
	42M43M	CGGCAAGACGGGGAGACCCC

*gyrA*	GF240	GGGTGATGTGATTGGTAAATACCAC
	GR319	TTCGAAAAATCTTGCGCCATTCTC
	87WT	CCATGGCGATAATGCGGTTTA
	87WC	CCATGGCGATAACGCGGTTTA
	87MA	CCATGGCGATAAAGCGGTTTA
	87MG	CCATGGCGATAAGGCGGTTTA
	91W	GCGGTTTATGATGCRCTAG
	91MG	TGCGGTTTATGGTGCRCTAG
	91MA	TGCGGTTTATAATGCRCTAT

	Quality control ^e^	TTTTTTTTTTTTTTTTTTTT

^a^ F: forward primer;  ^b^ R: reverse primer; reverse primers used for microarray have biotin conjugated at 5'-ends;  ^c^ W: wild genotype probe;  ^d^ M: mutant genotype probe, an oligonucleotide of 12 T's with an amino-labeled 3'-end was conjugated to the 3'-ends of all probes;  ^e^ an oligonucleotide of 20 T's with an amino-labeled 3'-end, biotin-labeled 5'-end was used as microarray quality control.

**Table 2:** Microarray and sequencing results of *CYP2C19∗2*.

*CYP2C19∗2*	Sequencing			
	AA	AG/GA	GG	Total
Microarray				
AA	82	2	1	85
AG/GA	6	507	29	542
GG	1	5	450	456
Total	89	514	480	1083

A sensitivity of 97.68%, specificity of 93.75%, positive predictive value of 93.94%, and negative predictive value of 98.68% for microarray detection of *CYP2C19∗2.*

**Table 3:** Microarray and sequencing results of *CYP2C19∗3*.

*CYP2C19∗3*	Sequencing			
	AA	AG/GA	GG	Total
Microarray				
AA	7	0	0	7
AG/GA	0	41	1	42
GG	0	11	1023	1034
Total	7	52	1024	1083

A sensitivity of 81.36%, specificity of 99.90%, positive predictive value of 97.96%, and negative predictive value of 98.94% for microarray detection of* CYP2C19∗3*.

**Table 4:** Microarray and sequencing results of *23S rRNA*.

*23S rRNA *(A2142G, A2143G)	Sequencing		
	GA/AG/GG	AA	Total
Microarray			
GA/AG/GG	255	14	269
AA	11	411	422
Total	266	425	691

A sensitivity of 95.86%, specificity of 96.71%, positive predictive value of 94.80%, and negative predictive value of 97.39% for microarray detection of *23S rRNA*.

**Table 5:** Microarray and sequencing results of *gyrA* at Asn-87.

*gyrA* N87K	Sequencing		
	A/G	T/C	Total
Microarray			
A/G	123	10	133
T/C	12	546	558
Total	135	556	691

A sensitivity of 91.11%, specificity of 98.20%, positive predictive value of 92.48%, and negative predictive value of 97.85% for microarray detection of *gyrA* at Asn-87.

**Table 6:** Microarray and sequencing results of *gyrA* at Asp-91.

*gyrA* D91G/ D91N	Sequencing		
	GG/AA	GA	Total
Microarray			
GG/AA	52	7	59
GA	5	627	632
Total	57	634	691

A sensitivity of 91.23%, specificity of 98.90%, positive predictive value of 88.14%, and negative predictive value of 99.21% for microarray detection of *gyrA* at Asp-91.

**Table 7:** Correlation between *H. pylori* mutations and CYP2C19 genetic polymorphism.

CYP2C19 polymorphism (cases)	Mutation rates (%) and cases (n)		
	*23S rRNA*	*gyrA* N87K	*gyrA* D91G/ D91N
Hom-EMs (284)	27.82 (79)	19.01 (54)	6.34 (18)
Het-EMs (343)	30.90 (106)	21.57 (74)	9.91 (34)
PMs (64)	17.19 (11)	15.63 (10)	6.25 (4)
*P *value	0.079	0.479	0.224

Hom-EMs: homozygous extensive metabolizers; Het-EMs: heterozygous extensive metabolizers; PMs: poor metabolizers

## Data Availability

The data used to support the findings of this study are available from the corresponding author upon request.

## References

[1] Liu G., Xie J., Lu Z. R. (2017). Fifth Chinese national consensus report on the management of Helicobacter pylori infection. *Zhonghua Nei Ke Za Zhi*.

[2] Nishizawa T., Suzuki H. (2015). Gastric carcinogenesis and underlying molecular mechanisms: helicobacter pylori and novel targeted therapy. *BioMed Research International*.

[3] Malfertheiner P., Megraud F., O'Morain A C. (2017). Management of Helicobacter pylori infection-the Maastricht V/Florence Consensus Repor. *Gut*.

[4] Biernat M. M., Iwańczak B., Bińkowska A., Grabińska J., Gościniak G. (2016). The prevalence of helicobacter pylori infection in symptomatic children: a 13-year observational study in the lower silesian region. *Advances in Clinical and Experimental Medicine*.

[5] Chen P.-Y., Wu M.-S., Chen C.-Y. (2016). Systematic review with meta-analysis: the efficacy of levofloxacin triple therapy as the first- or second-line treatments of Helicobacter pylori infection. *Alimentary Pharmacology & Therapeutics*.

[6] Georgopoulos S. D., Papastergiou V., Karatapanis S. (2015). Treatment of Helicobacter Pylori infection: optimization strategies in a high resistance era. *Expert Opinion on Pharmacotherapy*.

[7] Kuo C.-H., Lu C.-Y., Shih H.-Y. (2014). CYP2C19 polymorphism influences Helicobacter pylori eradication. *World Journal of Gastroenterology*.

[8] Lin Y.-A., Wang H., Gu Z.-J. (2017). Effect of CYP2C19 gene polymorphisms on proton pump inhibitor, amoxicillin, and levofloxacin triple therapy for eradication of Helicobacter Pylori. *Medical Science Monitor*.

[9] Shin J. M., Kim N. (2013). Pharmacokinetics and pharmacodynamics of the proton pump inhibitors. *Journal of Neurogastroenterology and Motility*.

[10] Li-Wan-Po A., Girard T., Farndon P., Cooley C., Lithgow J. (2010). Pharmacogenetics of CYP2C19: functional and clinical implications of a new variant CYP2C19^∗^17. *British Journal of Clinical Pharmacology*.

[11] Kuo C.-H., Kuo F.-C., Hu H.-M. (2012). The optimal first-line therapy of *Helicobacter pylori* Infection in year 2012. *Gastroenterology Research and Practice*.

[12] Peng X., Song Z., He L. (2017). Gastric juice-based real-time PCR for tailored Helicobacter Pylori treatment: a practical approach. *International Journal of Medical Sciences*.

[13] Kargar M., Ghorbani-Dalini S., Doosti A., Souod N. (2012). Real-time PCR for Helicobacter pylori quantification and detection of clarithromycin resistance in gastric tissue from patients with gastrointestinal disorders. *Research in Microbiology*.

[14] Nishizawa T., Suzuki H., Hibi T. (2009). Quinolone-based third-line therapy for Helicobacter pylori eradication. *Journal of Clinical Biochemistry and Nutrition*.

[15] Nishizawa T., Suzuki H., Kurabayashi K. (2006). Gatifloxacin resistance and mutations in gyrA after unsuccessful Helicobacter pylori eradication in Japan. *Antimicrobial Agents and Chemotherapy*.

[16] Teh X., Khosravi Y., Lee W. C. (2014). Functional and molecular surveillance of Helicobacter pylori antibiotic resistance in Kuala Lumpur. *PLoS ONE*.

[17] Miftahussurur M., Syam A. F., Nusi I. A. (2016). Surveillance of Helicobacter pylori antibiotic susceptibility in Indonesia: different resistance types among regions and with novel genetic mutations. *PLoS ONE*.

[18] Liou J.-M., Wu M.-S., Lin J.-T. (2016). Treatment of Helicobacter pylori infection: where are we now?. *Journal of Gastroenterology and Hepatology*.

[19] Martos M., Bujanda L., Salicio Y. (2014). Clarithromycin for first-line treatment of Helicobacter pylori infection after culture in high-resistance regions. *European Journal of Gastroenterology & Hepatology*.

[20] Cammarota G., Cianci R., Cannizzaro O. (2000). Efficacy of two one-week rabeprazole/levofloxacin-based triple therapies for *Helicobacter pylori* infection. *Alimentary Pharmacology & Therapeutics*.

[21] Glocker E., Stueger H.-P., Kist M. (2007). Quinolone resistance in *Helicobacter pylori* isolates in Germany. *Antimicrobial Agents and Chemotherapy*.

[22] Matsumoto Y., Miki I., Aoyama N. (2005). Levofloxacin- versus metronidazole-based rescue therapy for H. pylori infection in Japan. *Digestive and Liver Disease*.

[23] Beckett C. G., Tjaden J., Burgess T. (2011). Evaluation of a prototype dengue-1 DNA vaccine in a Phase 1 clinical trial. *Vaccine*.

[24] Han R., Lu H., Jiang M.-W. (2016). Multicenter study of antibiotic resistance profile of* H. pylori* and distribution of CYP2C19 gene polymorphism in rural population of Chongqing, China. *Gastroenterology Research and Practice*.

[25] Ieiri I., Kubota T., Urae A. (1996). Pharmacokinetics of omeprazole (a substrate of CYP2C19) and comparison with two mutant alleles, *CYP*2*C*19_*m*1_ in exon 5 and *CYP*2*C*19_*m*2_ in exon 4, in Japanese subjects. *Clinical Pharmacology & Therapeutics*.

[26] Jin D., Qi H., Chen S., Zeng T., Liu Q., Wang S. (2008). Simultaneous detection of six human diarrheal pathogens by using DNA microarray combined with tyramide signal amplification. *Journal of Microbiological Methods*.

[27] Zhang Y., Liu Q., Wang D., Chen S., Wang S. (2013). Simultaneous detection of oseltamivir- and amantadine-resistant influenza by oligonucleotide microarray visualization. *PLoS ONE*.

[28] Zhang J., Zhong J., Ding J. (2018). Simultaneous detection of human CYP2C19 polymorphisms and antibiotic resistance of Helicobacter pylori using a personalised diagnosis kit. *Journal of Global Antimicrobial Resistance*.

